# Sensationalism in Public Performances

**Published:** 1889-01-26

**Authors:** David Walsh


### Sensationalism in Public Performances.

By David Walsh, L.R.C.P. and S., Edin.

Probably one of the earliest amusements of mankind has been the witnessing of feats of skill and strength, in which there is almost always more or less risk to the performers. The element of brutality, however, may be said to have steadily diminished before the growth of that moral feeling which recognises the sacredness of human life. As a proof of this tendency, one may compare the national sports of three countries at different periods. First, our own prize-fighting of a generation ago, which was rarely fatal to the pugilists; next, the bull-fighting of Spain, in which life has been steadily sacrificed for centuries; and thirdly, the gladiatorial combats of ancient Rome, when, except by the merest chance, death was a certainty for one or both of the combatants. So that the broad generalisation may be laid down that where life is held in light esteem, the public amusements will be correspondingly brutal, and *vice versa*. And yet, notwithstanding all this, the inhabitants of Great Britain in the Victorian era turn out daily in tens of thousands to see feats which hazard the lives of performers hardly less than was the case in the arenas of Madrid or Rome.

This regard for the sacredness of life, which may be reckoned among the chief characteristics of modern times, is also extended to the lower animals. There are societies for the protection of horses, dogs, cats, pigeons, and other domestic animals. There are laws which forbid a scientific man to touch any of these creatures with a knife, even though his experiment be planned with the highest ends in view of which the mind is capable. Yet, in spite of this organised sentiment—this refined humanity—the fact remains that men, women, and children are allowed to risk their lives daily in pandering to the popular taste for sensational amusement. It is a curious inconsistency that a man, however learned and humane, should not be permitted to carry out a well-reasoned experiment, say, on a cat, without making himself liable to fine and imprisonment; while a circus proprietor, with the sordid motive of filling his own pocket, may engage his acrobats night after night in performances that are simply playing fast and loose with death. Should the unlucky tumbler break his neck, there is no question as to liability for damages on the part of his employer; and a clause to provide for cases of this kind might well be inserted in the new Bill that is shortly to come before Parliament.

In discussing this matter, one should not lose sight of the fact that there is hardly any pursuit calling for the exercise of strength and skill which does not involve some amount of risk. An accident is an unforeseen occurrence, and is therefore unavoidable; but the dangers of a performance may be increased or invited to an extent that, from a moral point of view, may become criminal. If a man walk on a rope stretched a few feet from the ground, a fall will do him little or no harm; but raise the stage a hundred feet, and the same "accident" will infallibly cause his death. As to attractiveness, where one person would come to see him near the ground, fifty would flock to the more dangerous performance. Indeed, it may be laid down as an axiom that the number of spectators will be in direct proportion to the peril of any given performance. Riding on horseback affords an instance of how a position of comparative safety may be artificially turned into one of extreme danger. A ride in Rotten Row or out in the country lanes is, of course, never quite safe from accidents; but these are trifling and reasonable compared with the chances of following the hounds across country. This sport, again, is infinitely less hazardous than steeplechasing, which is by far the most fatal form of horse-riding. The same question of degree is shown in the case of the circus rider, who is safe enough on his bare-backed steed; but if, by making the animal rear bolt upright time after time, the chances of his being killed are very much increased, then the performance becomes barbarian. To draw the line at the exact point where the courting of danger becomes unjustifiable would be no easy matter; but, on the other hand, many cases exist in which there could not be a moment's doubt. The question then arises whether Government should interfere, seeing that one of its first duties is to protect the subject in life and limb. Acting on that principle, such practices as prize-fighting and duelling have been put a stop to; and many worthy people maintain that steeplechasing should be suppressed in the same way. The chief argument in favour of the latter sport is that it tends to keep up the breed of horses; but surely that end could be attained by less disastrous means. No adequate object, however, can be claimed for the reckless feats commonly shown in public; and most candid men will agree that their discontinuance would be a direct gain to the community.

The assertion, already made, that this needless imperilling of life is of daily occurence requires little proof. It is hardly overstating the case to say that almost every mixed acrobatic performance of any size introduces at least one or two feats in which lives are endangered. Readers will readily recall instances of the kind, either from hearsay or from actual experience. Some may remember when the famous Blondin began walking on the high rope, that is to say, on a cable tightly stretched at a great height from the ground. The daring acrobat seemed quite at home on his perch, where he cut all kinds of capers, such as pretending to fall and catch himself, drawing up a stove to cook an omelet, and walking over blindfold. All this was dangerous enough, as a fall from such a height must have been fatal; but this performer did not stop at personal risk, for he used not only to carry a man over on his back, but actually to wheel him across in a wheel-barrow. Blondin retired with a fortune long ago, but lately has resumed his old occupation. His nerves seem still to be of iron, and it would be a thousand pities if he were to meet with a misfortune after escaping thus far; still the old adage about the pitcher getting broken at last is nowhere more certain of application than in these risky performances. This veteran may be called the King of Acrobats. There is a magnificence about his feats that shows what is possible to the trained nerve and muscle of that splendid animal—man. Blondin crossing between the towers of the Crystal Palace, or in that climax of daring, the walk over the rapids of Niagara, is a spectacle to make one ponder and marvel.

Here is a little nineteenth century picture taken from a *Daily News* telegram: "At Bone, in Algeria, an exciting scene occurred lately at the Aquarium—a sort of itinerant menagerie. A special feature consisted in the public feeding of no fewer than 70 crocodiles by the manager, M. Pernolet. On this occasion he was sitting on the back of his largest crocodile, and was feeding the rest, when all at once, as he turned his head and put out his hand to the attendant for a piece of meat, one of the others crawled up to him and bit him in the stomach. A shout was raised by the spectators, and those around the tank tried to beat away the crocodile, who, notwithstanding M. Pernolet's blow, began whirling round his prey as if to tear him to pieces. Unfortunately, in struggling, M. Pernolet slipped, and fell in the very midst of the reptiles, which all rushed on him with fury. A panic took place among the spectators, who mostly fled. Nevertheless M. Pernolet was rescued, and although his wounds are serious, his life is not thought to be in danger."

In our own country a common sensational feat is diving from a high roof into a net, turning one or more somersaults in the descent. A clumsy fall or a defective netting in this case would mean death. In another trick a woman is caught in the same way after seemingly being shot out of a cannon, but in reality she runs very little risk; indeed, the danger of these performances is often more apparent than real. Had Blondin, for instance, devoted himself to lion encounters instead of walking through mid-air at perilous heights, there can be little doubt he would never have reached his present age. Then, again, on the other hand, many simple-looking tricks are extremely hazardous. Some friends of the present writer went into a show-tent pitched on the sands of a French watering-place. Among other feats a "strong man" was in the habit of lifting a cannon on to his shoulder and firing it off; but on this occasion he sank to the ground beneath its weight and was literally crushed to death. The unfortunate stroller went through his performances many times daily, and the weather being hot, he had exhausted his strength by swimming in the sea between whiles, so that in the end he broke down and perished miserably.

Balloon ascents have always been beloved of the populace, partly because of their comparative rarity, and partly, as it would seem, from their extreme danger. A hundred things may happen: the balloon may burst; the valves go wrong; and there are the manifold chances of the descent. Altogether, the odds are not strongly in favour of the voyagers being seen again alive. But most of the mishaps mentioned are remote; the public prefer something to be done before their eyes, a demand which caterers have done their best to meet. Some time back a woman caused a great sensation by hanging on to the car of an ascending balloon—so it was given out—by her teeth. In answer to a question, however, which was put in the House of Commons, it was stated that the acrobat was securely fastened to the car in other ways, reminding one of the account given in Sir Walter Scott's novel, "Fair Maid of Perth," of the hanging of Bonthron. That worthy swung from the gallows without harm, supported by a cunning contrivance of slings and bandages, until his friends could carry him away. But the glory of balloon ascents, however managed, has of late been quite eclipsed by sensational methods of descent. An American has devised a parachute—the first that has not promptly killed its inventor—which lands him safely on the bosom of mother earth. The machine expands in coming down, and the slightest hitch in apparatus or flaw in materials would cause the death of the operator. Meanwhile, people of all sorts and conditions flock in crowds to see the performance of this bold aëronaut, and bring him and his employers a golden harvest. This reckless American surely merges bravery into foolhardiness, and although he may be the pioneer of a great future in aërial navigation, yet a single descent in safety would have proved his point sufficiently for all scientific purposes.

Some day those worthy people who now devote their attention to the protection of the lower animals may be induced to look after the welfare of their fellow-creatures a little more closely. A tithe of their energies would soon force Government to forbid these dangerous performances, both by direct penalties and by imposing a heavy employer's liability. Quite lately a fatal accident took place in a London exhibition. To quote an eye witness, "the injured man was carried out neck and heel like a dead sheep, and all the attendants seemed to care for was to get him out of sight." As a matter of fact the unfortunate acrobat's thigh was broken, and he died soon afterwards. The secretary of the management pleaded that "the man could hardly be treated in the arena, before thousands of persons." In other words, these people who had come to see a frightfully hazardous performance could not be put to the discomfort of witnessing its results. So that the unlucky victim was dragged out of their sight, a living sacrifice to sensationalism, and deprived even of the small comfort of having his broken bones fastened up to prevent further pain and injury. Here is a little play, a tragedy in one act, with the public, the performer, and the secretary as *dramatis personæ*. The public pays abundant gate-money, and watches events with eager interest. The performer pays his life, and his mangled body is at once carried off by a troop of fellow-servants. The secretary pays whom he likes, usually nothing beyond wages due up to time of accident. There is one other personage wanted to fill up the measure of poetical justice, and that is the Public Prosecutor.

